# Cryopreservation of mouse thymus depletes thymocytes but supports immune reconstitution on transplantation

**DOI:** 10.1002/eji.202350546

**Published:** 2023-10-17

**Authors:** Mira M. Chawda, Susan Ross, Ching‐In Lau, Diana C. Yánez, Jasmine Rowell, Peter Kilbride, Tessa Crompton

**Affiliations:** ^1^ UCL Great Ormond Street Institute of Child Health London UK; ^2^ Cytiva, Danaher Corporation Cambridge UK

**Keywords:** Clinical immunology, Cryopreservation, Thymopoiesis, Thymus transplantation

## Abstract

Cryopreservation of mouse thymus depletes donor thymocytes but preserves thymus function when transplanted after thawing into athymic mice. No differences in immune reconstitution were observed between fresh and frozen/thawed transplants suggesting that donor thymocyte depletion does not affect outcome. Thus, cryopreservation of thymus may improve outcomes in thymus transplant patients.

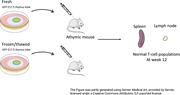

The thymus is essential for T‐cell development, so athymic infants cannot produce T‐cells and lack protection against infectious diseases. Cortical(c) thymic epithelial cells (TEC) are required for T‐cell fate induction and positive selection of the TCR repertoire and medullary(m)TEC are required for tolerance induction [1]. Treatment of athymic patients by transplantation of thymus tissue provides the indispensable environment for T‐cell development, so progenitor cells migrate from recipient bone marrow into the transplanted tissue, where donor TEC signal for T‐cell fatespecification and development, and recipient T cells are produced [2, 3].

Thymus is removed during pediatric cardiac surgery, cut into ∼1mm‐deep slices, and cultured for 2–3 weeks to partially deplete donor thymocytes (which could cause graft‐versus‐host‐disease) before transplantation [2, 3]. Although this procedure is life‐saving, recipients have low T‐cell counts, some develop autoimmune diseases, and it is sometimes unsuccessful [2, 3]. MHC‐matching between donor and recipient is not currently possible because of the urgency of treatment, so freezing the thymus for transplantation would have many advantages: increasing availability of thymus transplantation; decreasing delays in treatment; preventing waste of tissue; providing treatment locally; and enabling MHC‐matching, which should improve immune reconstitution and reduce autoimmunity.

Cryopreservation of tissues for transplantation is challenging [4]. Heat transfer, ice nucleation, intracellular ice formation, cryoprotective agent, and cooling protocols, all impact on viability/function of thawed tissue [4, 5, 6]. Additional complexity arises from the heterogeneity of cell types present in tissues, which may have different requirements for preservation. These differences in freezing/thawing could be harnessed to deplete unwanted cells from tissues for transplantation [7]. For the thymus, it would be beneficial to avoid the long culture period that is used for thymocyte depletion, because it can lead to necrosis [8].

We showed that human thymus slices can induce and support mouse T‐cell development after cryopreservation when transplanted into athymic mice [9]. It was not possible to investigate the impact of freezing on the survival of donor thymocytes/T‐cells because human T‐cells cannot survive in nude mice [10, 11]. Here we investigate the impact of cryopreservation on thymus function following transplantation by introduction of MHC‐matched mouse thymus into nude (athymic) mice to test if freezing can deplete the thymus of T‐cells while preserving TEC function.

Thymocytes are susceptible to induction of cell death by several pathways [12], so we measured the impact of freeze/thaw on cell viability in embryonic day (E)17.5 fetal thymus organ culture (FTOC), under conditions that maintain TEC function [9]. One lobe from each thymus was frozen, thawed, and cultured (cryopreserved lobe (CPL)) ([9, 13–15], Supporting information Methods), while the second control noncryopreserved lobe (NCPL), was cultured fresh. Freeze/thaw efficiently depleted thymocytes. On day(D)0 after thawing there was a 94.5% reduction in viable cells in CPL compared with NCPL, which rose to >99.0% on D4 (Fig. 1A).

**Figure 1:**
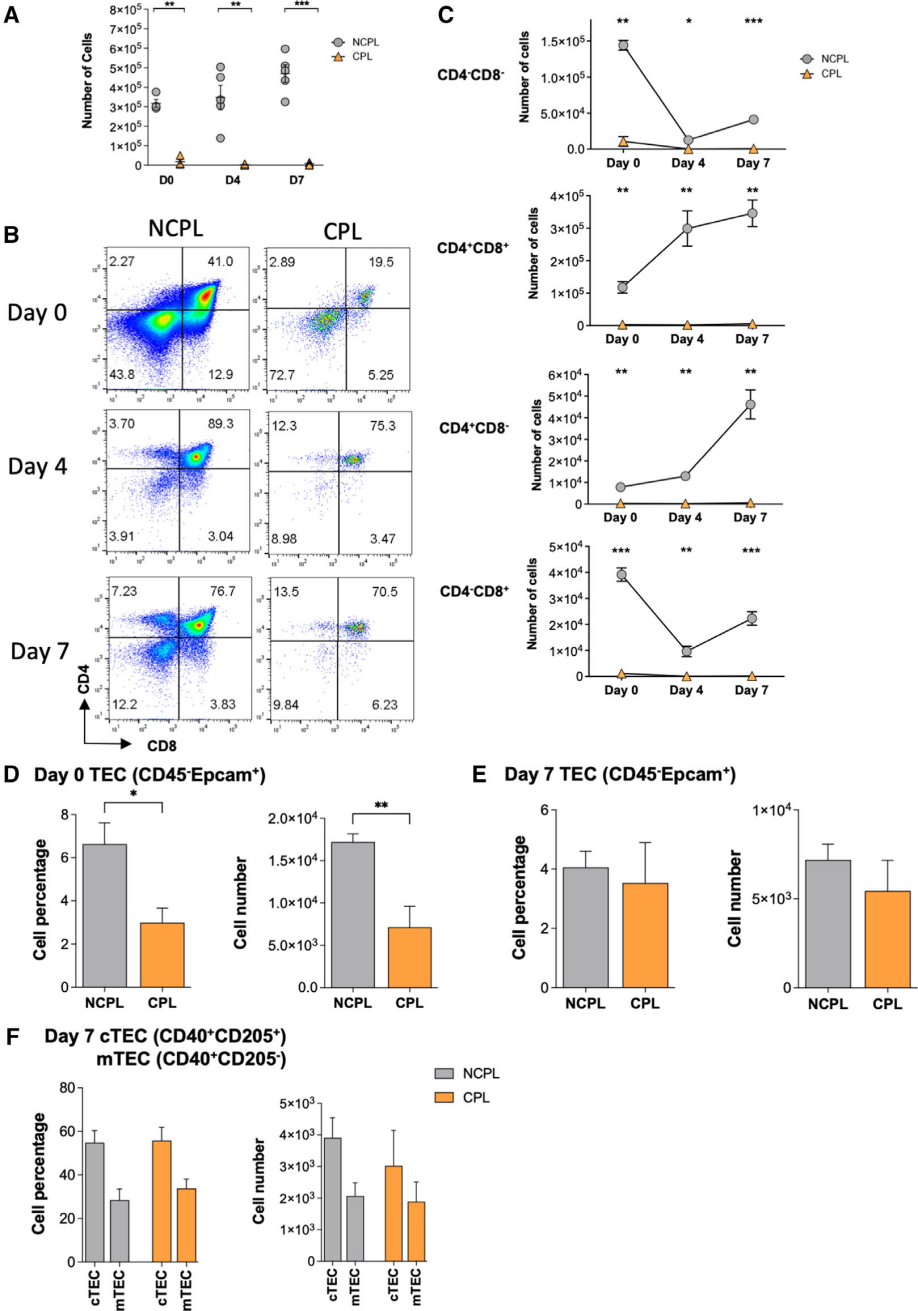
Freezing and thawing deplete the thymus of developing T‐cells. One lobe from E17.5 C57BL/6 thymus was cultured in FTOC fresh (NCPL, grey) or after freeze/thaw (CPL, orange), and analyzed by flow cytometry on Day(D)0 (*n* = 4), D4 (*n* = 5), and D7 (*n* = 5). Data representative of three independent experiments. (A) Scatter‐plot: number of cells on D0, D4, and D7. Each shape represents an individual lobe. (B) Flow cytometry (gating in Supporting information Fig. S1A): CD4 versus CD8, D0, D4, and D7. (C) Plots: number of cells in CD4‐CD8−, CD4+CD8+, CD4+CD8−, and CD4‐CD8+ populations on D0, D4, and D7. (D) Bar charts: percentage and number of TEC(CD45‐Epcam+) D0. (E) Bar charts: percentage and number of TEC(CD45‐Epcam+) D7. (F) Bar charts: percentage and number of cTEC(CD45‐Epcam+CD205+CD40+) and mTEC(CD45‐Epcam+CD205−CD40+) D7. Mean ± SEM, Student's *t*‐test. *P < 0.05, **P < 0.01, ***P < 0.001.

During T‐cell development, CD4‐CD8‐double‐negative (DN) progenitors differentiate to the CD4+CD8+ double‐positive (DP) stage, through a CD8+ immature single‐positive (ISP) intermediate. The DP population gives rise to mature CD4SP and CD8SP cells, following TCR engagement by MHC+peptide on TEC [1]. On E17.5, the mature SP and mTEC populations have not developed, and ∼40% of cells are DP [13, 14, 15]. On D0 NCPL FTOC contained 41%DP cells, and 12.9%ISP (Fig. 1B). In the CPL ∼73% of cells were DN, suggesting this population contained more cryo‐resistant cells (Fig. 1B). The NCPL showed the normal pattern of T‐cell development (Fig. 1B, C). The CPL also showed an increase in DP and SP cells on D7 (Fig. 1B), but cell numbers were reduced at all timepoints in all thymocyte populations in CPL compared with NCPL (Fig. 1B, C). In contrast, on D0 the proportion and number of TEC was reduced by only ∼2‐fold in CPL compared with NCPL (Fig. 1D), and on D7 we detected no differences in TEC cell number overall (Fig. 1E), or in cTEC and mTEC number between NCPL and CPL, indicating that TEC survived and differentiated following freeze/thaw (Fig. 1E, F, Supporting information Fig. S1B, C).

Given this efficient depletion of thymocytes and TEC survival, we investigated the impact of cryopreservation on donor and recipient T‐cell populations in vivo, after transplant into a congenic nude mouse ([9, 10], Supporting information Methods). We transplanted sex‐matched E17.5 GFP‐transgenic thymus, so the only factor that might influence immune reconstitution and donor T‐cell survival would be freezing/thawing. GFP distinguished between donor (GFP+) and recipient (GFP−) T‐cells in peripheral blood following transplantation (Fig. 2A–D, SFig. 1D). From Week (W) 2, GFP+ cells were detected in the blood of both groups. Percentages of GFP+ and CD3+GFP+ cells were higher in the NCPL‐transplanted than the CPL‐transplanted blood on W6 and W8 (Fig. 2A, B). The percentage of GFP+CD4+CD3+ T‐cells rose to peak at W4‐6, and was higher in the NCPL‐transplant than CPL‐transplant group on W6 and W8, whereas the GFP+CD8+CD3+ T‐cell populations showed no differences between groups (Fig. 2D). The percentage of GFP‐CD4+CD3+ and GFP‐CD8+CD3+ T‐cells increased steadily, but were not different between groups (Fig. 2C, D). Comparison between donor and recipient populations showed that for CD4+CD3+ T‐cells, both NCPL and CPL groups had higher percentages of donor than recipient cells at W2, but from W8‐12 the NCPL had higher percentages of recipient cells (Fig. 2C). In contrast, the CD8+CD3+ population in the NCPL group had higher percentages of donor cells on W4 and W6, but we found no differences between groups from W8‐12 (Fig. 2D).

**Figure 2:**
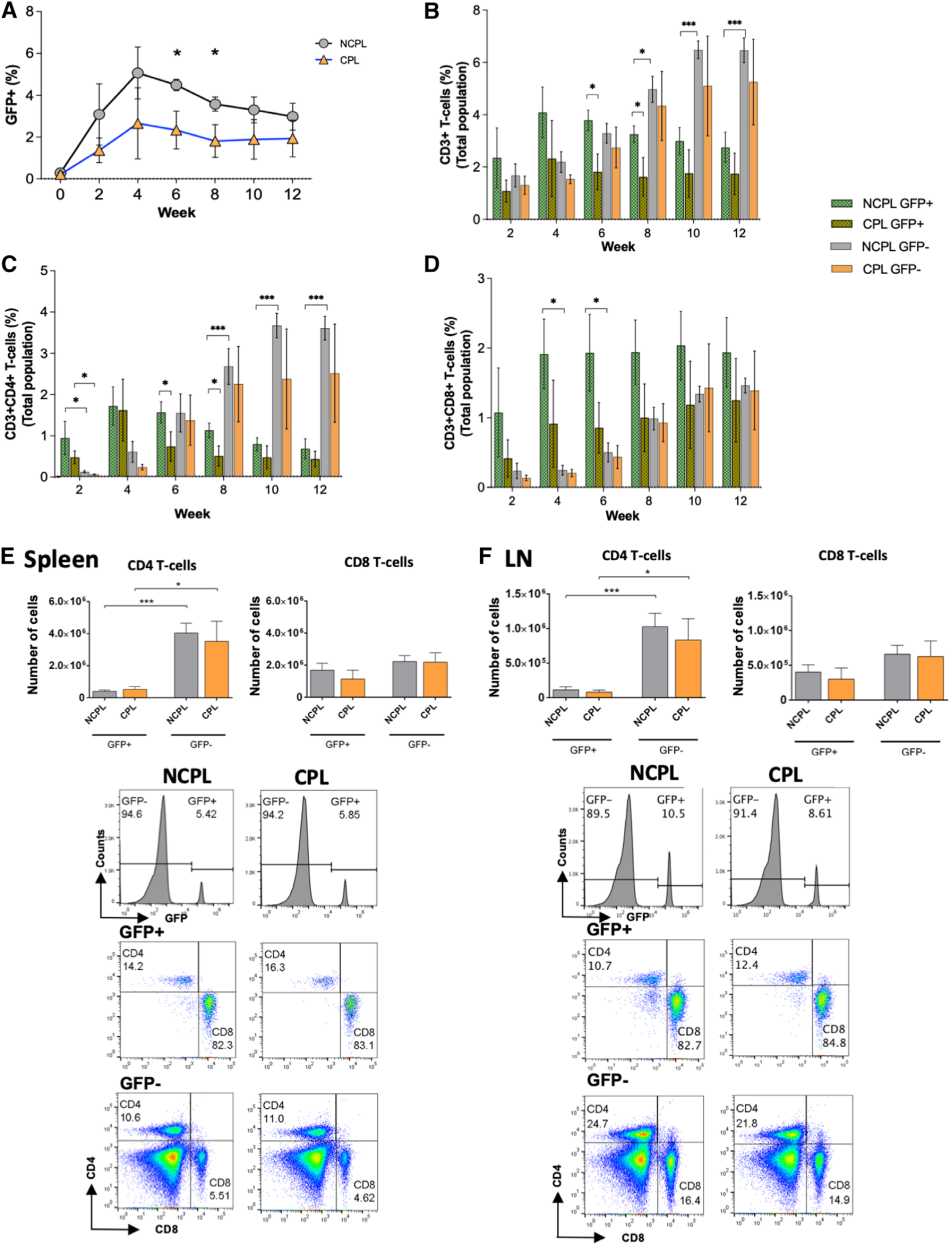
Immune reconstitution in blood, spleen, and LN of nude recipients that received fresh or frozen/thawed thymus transplants. C57BL/6 nude mice transplanted subcutaneously with three congenic sex‐matched GFP‐tg E17.5 thymus lobes fresh (NCPL, grey, *n* = 5) and after freeze/thaw (CPL, orange, *n* = 5). Data representative of three independent experiments. (A–D) Blood was analyzed by flow cytometry on Week (W) of transplant (W0) and at 2‐week intervals to W12. (A) Plot: percentage GFP+ cells (gating in Supporting information Fig. S2). (B, D) Bar‐charts: percentage NCPLGFP+(green), CPLGFP+(brown), NCPLGFP−(grey), CPLGFP−(orange) cells: CD3+ (B), CD3+CD4+ (C), CD3+CD8+ (D). (E, F) W12 Spleen (E), LN (F). Bar charts: number of CD4 and CD8 T‐cells. Histograms: GFP‐fluorescence. Plots: CD4 and CD8, gated on TCRβ+, in GFP+(upper), and GFP−(lower). Mean±SEM, Student's *t‐*test. *P < 0.05, **P < 0.01, ***P < 0.001.

On sacrifice (W12), thymus tissue recovered from the site of transplant showed normal GFP− T‐cell development, as described previously [10], and contained <0.45% GFP+ thymocytes (Supporting information Fig. S2). In spleen and LN, most GFP+ cells were CD8+ in both groups (Fig. 2E, F). There were no differences in number of GFP+CD4+ or GFP‐CD4+ T‐cells between NCPL and CPL groups, and both contained on average ∼10‐fold more GFP‐CD4+ than GFP+CD4+ T‐cells (Fig. 2E, F). There were no differences in number of GFP+CD8+ and GFP‐CD8+ T‐cells within or between either group. We detected no differences in the number of recipients or donors γδT‐cells, NKT‐cells, and NK cells, between groups in the spleen or LN (Supporting information Fig. S3). The number of donor NK cells was very low (on average: spleen 3000 and 3500, LN 600 and 440 for NCPL and CPL, respectively; Supporting information Fig. S3).

Overall, we showed that mouse thymus that has been frozen/thawed can support T‐cell development when transplanted into nude mice to the same extent as fresh thymus and that although freeze/thaw rapidly killed developing T‐cells, and led to subtle changes in dynamics of reconstitution observed in blood, it had no impact on peripheral immune reconstitution. This suggests that donor T‐cells found on sacrifice in peripheral lymphoid tissues were produced in the donor thymus after transplantation, and therefore that thymocyte depletion (by cryopreservation or 2–3‐week culture), is likely to have little impact on outcome. This paves the way for MHC‐matching between recipient and donor, as the use of MHC‐matched cryopreserved tissue is likely to improve patient outcomes. Interestingly, analysis of peripheral lymphoid organs revealed clear differences in the reconstitution of CD4 and CD8 populations. The CD4 compartment contained >90% recipient T‐cells, whereas the CD8 compartment contained similar numbers of donor and recipient T‐cells, suggesting that donor CD8 T‐cells expanded in lymphopenic hosts to contribute more to the peripheral pool.

## Author contributions

Mira M. Chawda, Susan Ross, Ching‐In Lau, Diana C. Yánez, and Jasmine Rowell contributed to the experiments. Tessa Crompton, Susan Ross, and Peter Kilbride helped with the funding. Mira M. Chawda, Ching‐In Lau, Susan Ross, Peter Kilbride, and Tessa Crompton prepared the manuscript.

## Conflict of interest

The authors declare no conflict of interest.

**Abbreviations**
c: cortical
CPL: cryopreserved lobe
E: embryonic day
FTOC: fetal thymus organ culture
GFP: green fluorescent protein
m: medullary
NCPL: nonCPL
TEC: thymic epithelial cell
W: week

## Supporting information

Supporting information

## Data Availability

The data that support the findings of this study are available from the corresponding author upon reasonable request.
