# Supplementary material for: Cryopreservation of mouse thymus depletes thymocytes but supports immune reconstitution on transplantation
Source: Eur J Immunol. 2023 Oct 17;53(12):2350546. doi: 10.1002/eji.202350546 (PMC10946610; doi:10.1002/eji.202350546)
Supplement: Supplementary file 1 — Supporting information [file EJI-53-0-s001.pdf]

Supplementary File: Materials and Methods

Mice

Mice were bred and maintained in specific pathogen-free conditions at University College London. Mouse studies were approved by the United Kingdom Home Office. All mice were on a C57BL/6 background. Ubiquitin-GFP-transgenic (GFP-tg) reporter mice[1], which express ubiquitous GFP under control of the human ubiquitin C promoter, and Nude mice were purchased from The Jackson laboratory. Recipients used for all experiments were age matched. For timed mates, two females were placed with a male overnight and the day of plug was counted as Embryonic day (E) 0.5.

Flow cytometry and cell sorting

Cells were stained as described [2] using antibodies from Biolegend: CD4-FITC (clone RM4-4; catalogue number 116004); CD4-PE (clone RM4-4; catalogue number 116006); CD3-PerCP/Cy5.5 (clone 17A2, catalogue number 100217); CD3-APC (clone 17A2, catalogue number 100236); CD8a-PerCP/Cy5.5 (clone 53-6.7; catalogue number 100734); TCRβ PE/Cy7 (clone H57-597, catalogue number 109221); CD45-APC/cy7 (clone 30-F11, catalogue number 103116); Epcam-BV421 (clone G8.8, catalogue number 118225); CD40-PE (clone 3/23, catalogue number 553791); TCR γ/δ-APC (clone GL3, catalogue number 118116); and from eBioscience: CD8a-PE (clone 53-6.7, catalogue number 12-0081-83); CD205-APC (clone 205yekta, catalogue number 17-2051-82); NK1.1-PE (clone PK136, catalogue number 12-5941-83). Data were acquired on C6 Accuri (BD Biosciences) and for TEC staining (Fig1D-F), samples were acquired on a CytoFLEX (Beckman Coulter). Data were analysed using FlowJo v10.5.3 (Tree Star, Ashland, OR, USA). Live cells were gated according to FSC/SSC profiles and doublets excluded by gating on FSC-H/FSCA. Fixable Viability Dye (VD) eFluor 660 (eBioscience) was used to exclude dead cells (Fig1), at a dilution of 1:100 according to the manufacturer's instructions.

Thymic epithelial cells (TEC) (CD45-Epcam+) were isolated as described[3, 4]; cortical (c)TEC and medullary (m) TEC were identified by staining against CD40 and CD205 (cTEC: CD40+CD205+; mTEC: CD40+CD205-)[3].

Fetal thymus organ culture (FTOC)

E17.5 thymus lobes were cultured as described[3-5].

Cryopreservation

Freezing was carried out as described[6]. In brief, each embryonic thymus lobe was placed in 1 ml of freezing media (7% DMSO (Thermo Fisher Scientific) in AIM-V medium) for 5 min on ice to allow the lobe to equilibrate. This freezing medium was removed and replaced with 12.5% DMSO in AIM-V medium [7]. The lobes were then immediately transferred to a pre-cooled controlled rate freezer (VIA Freeze Uno (Cytiva)) at 4°C and held for 10 min. The controlled rate freezer was then cooled at -1°C/min until reaching -80°C.

Sample thawing

Samples were removed from storage (-80°C freezer or LN_2 at the vapour phase) and thawed rapidly in a 37°C water bath. Then, sample medium were diluted in a stepwise manner to remove DMSO, by transferring the medium containing the thawed lobe into a 15 ml centrifuge tube containing 1 ml fresh AIM-V medium at room temperature, and leaving it to stand for 1 min. AIM-V medium was then added slowly until a volume of 12 ml was reached. The DMSO-containing medium was then removed, and the thawed thymic lobe was either placed into FTOC or used for in vivo transplants.

Thymus transplantation

Female GFP-tg mice were timed mated with WT C57BL/6 males to generate embryos that expressed the ubiquitous GFP reporter. Thymus was dissected on E17.5, and from each bi-lobed thymus one lobe was transplanted fresh (on the day of dissection) and one lobe was frozen to -80C and thawed on the same day for transplantation. Sex of embryos was determined by PCR as described[8]. Thymus lobes were transplanted into sex-matched nude recipients, as described[6, 9]. In brief, three litter-mate lobes of non-cryopreserved (NCPL, fresh) thymus or their cryopreserved counterparts (CPL) were pooled in 1x Phosphate Buffered Saline (PBS (HyClone)) and injected subcutaneously in scruff. The

thymic lobe preparation was injected subcutaneously into scruff of the neck of a nude mouse for the non-cryopreserved pool and into another age-matched nude mouse for the cryopreserved pool. Nude mice were injected at age 4-8 weeks. Baseline tail bleeds were taken at week 0 following the transplant. Tail bleeds were performed every two weeks, until 12 weeks post-transplant when recipients were sacrificed for analysis of spleen and inguinal lymph nodes.

Statistics

Unpaired two-tailed Student's t-tests were used as stated and probabilities considered significant if $p<0.05$(*), $p< 0.01$(**), and $p< 0.001$(***).

References

1 **Schaefer, B. C., Schaefer, M. L., Kappler, J. W., Marrack, P. and Kedl, R. M.,** Observation of antigen-dependent CD8+ T-cell/ dendritic cell interactions in vivo. *Cell Immunol* 2001. **214**: 110-122.

2 **Papaioannou, E., Yánez, D. C., Ross, S., Lau, C.-I., Solanki, A., Chawda, M. M., Virasami, A., Ranz, I., Ono, M., O'Shaughnessy, R. F. L. and Crompton, T.,** Sonic Hedgehog signaling limits atopic dermatitis via Gli2-driven immune regulation. *The Journal of Clinical Investigation* 2019. **129**: 3153-3170.

3 **Saldana, J. I., Solanki, A., Lau, C. I., Sahni, H., Ross, S., Furmanski, A. L., Ono, M., Hollander, G. and Crompton, T.,** Sonic Hedgehog regulates thymic epithelial cell differentiation. *J Autoimmun* 2016. **68**: 86-97.

4 **Lau, C. I., Barbarulo, A., Solanki, A., Saldana, J. I. and Crompton, T.,** The kinesin motor protein Kif7 is required for T-cell development and normal MHC expression on thymic epithelial cells (TEC) in the thymus. *Oncotarget* 2017. **8**: 24163-24176.

5 **Drakopoulou, E., Outram, S. V., Rowbotham, N. J., Ross, S. E., Furmanski, A. L., Saldana, J. I., Hager-Theodorides, A. L. and Crompton, T.,** Non-redundant role for the transcription factor Gli1 at multiple stages of thymocyte development. *Cell Cycle* 2010. **9**: 4144-4152.

6 **Ross, S., Cheung, M., Lau, C. I., Sebire, N., Burch, M., Kilbride, P., Fuller, B., Morris, G. J., Davies, E. G. and Crompton, T.,** Transplanted human thymus slices induce and support T-cell development in mice after cryopreservation. *Eur J Immunol* 2018. **48**: 716-719.

7 **Kilbride, P., Lamb, S., Milne, S., Gibbons, S., Erro, E., Bundy, J., Selden, C., Fuller, B. and Morris, J.,** Spatial considerations during cryopreservation of a large volume sample. *Cryobiology* 2016. **73**: 47-54.

8 **Rowbotham, N. J., Hager-Theodorides, A. L., Cebecauer, M., Shah, D. K., Drakopoulou, E., Dyson, J., Outram, S. V. and Crompton, T.,** Activation of the Hedgehog signaling pathway in T-lineage cells inhibits TCR repertoire selection in the thymus and peripheral T-cell activation. *Blood* 2007. **109**: 3757-3766.

9 **Furmanski, A. L., O'Shaughnessy, R. F., Saldana, J. I., Blundell, M. P., Thrasher, A. J., Sebire, N. J., Davies, E. G. and Crompton, T.,** T-cell reconstitution after thymus xenotransplantation induces hair depigmentation and loss. *J Invest Dermatol* 2013. **133**: 1221-1230.

Supplementary Figure 1

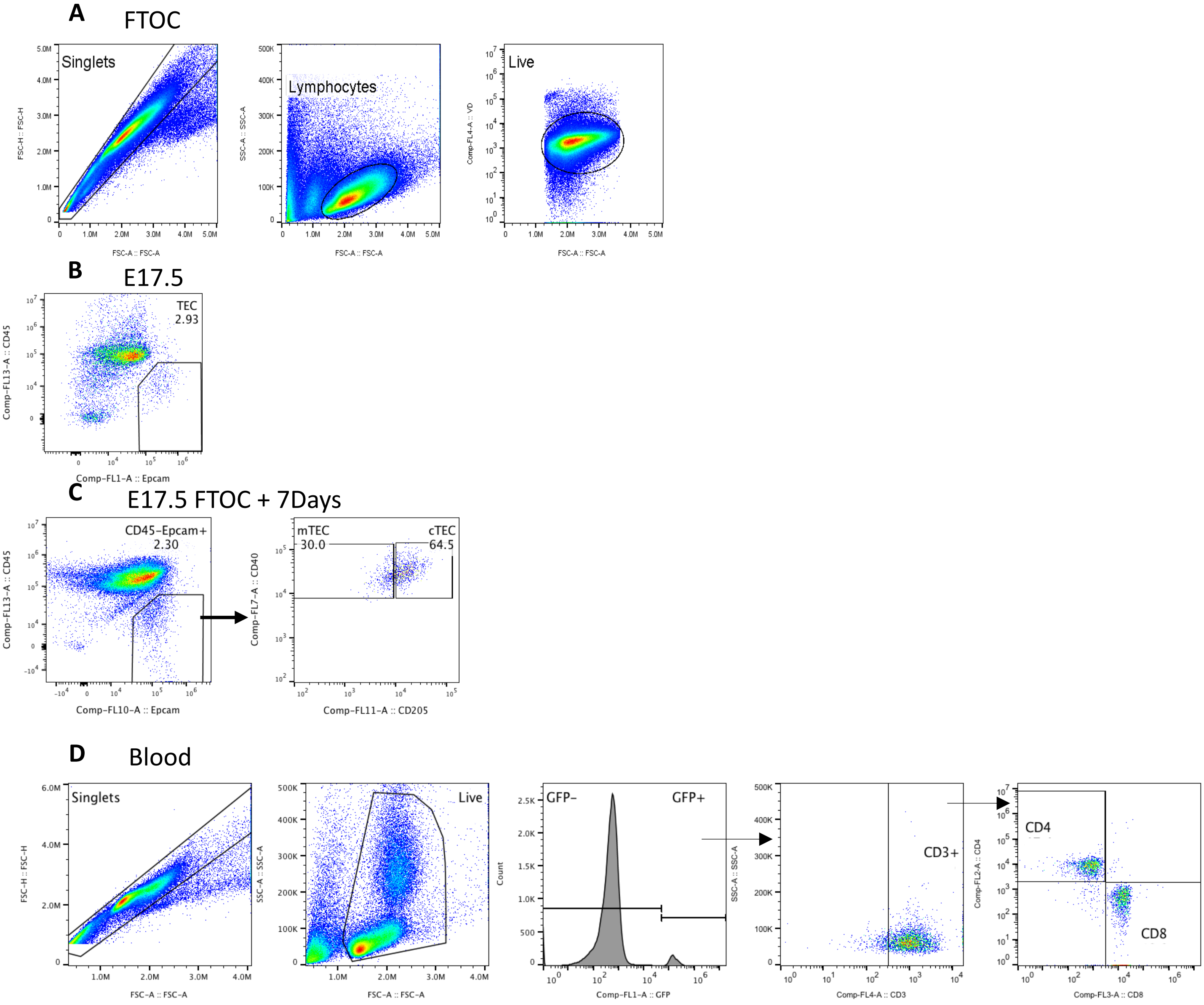

Supplementary Figure 1 Legend

(A) Flow cytometry plots show the strategy to gate on viable cells in FTOC. Events were first gated to exclude doublets, then gated by FSC-A and SSC-A, then gated by FSC-A against viability dye (VD) to exclude VD+ cells.

(B) Flow cytometry plot to show gating strategy to identify TEC (CD45-Epcam+) in FTOC.

(C) Flow cytometry plots to show gating strategy to identify cTEC (CD205+CD40+) and mTEC (CD205-CD40+) in FTOC D7 (first gated on CD45-Epcam+ (as in B)).

(D) Flow cytometry plots show the gating strategy to identify GFP+ and GFP- T-cells in blood. Events were first gated to exclude doublets, then gated by FSC-A and SSC-A to identify lymphocytes and leukocytes, then gated GFP+ (donor) and GFP- (recipient), then gated on CD3+ cells, before analysis of CD4 and CD8 expression.

Supplementary Figure 2

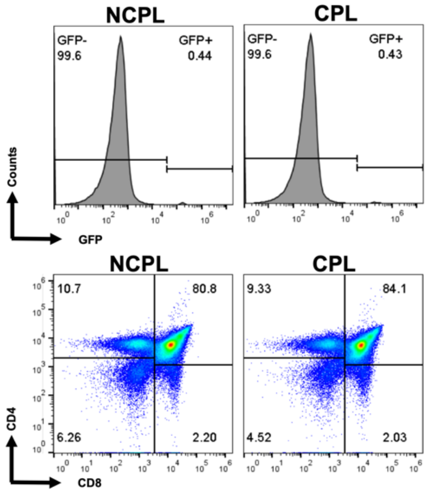

Supplementary Figure 2 Legend

Flow cytometry analysis of thymus tissue recovered from the site of transplant from NCLP (left) and CPL (right) groups. Histograms show GFP fluorescence, giving the percentage of cells in the markers shown. The lower flow cytometry plots show anti-CD4 and anti-CD8 staining, gated on GFP- cells, giving the percentage of cells in the quadrants shown.

Supplementary Figure 3

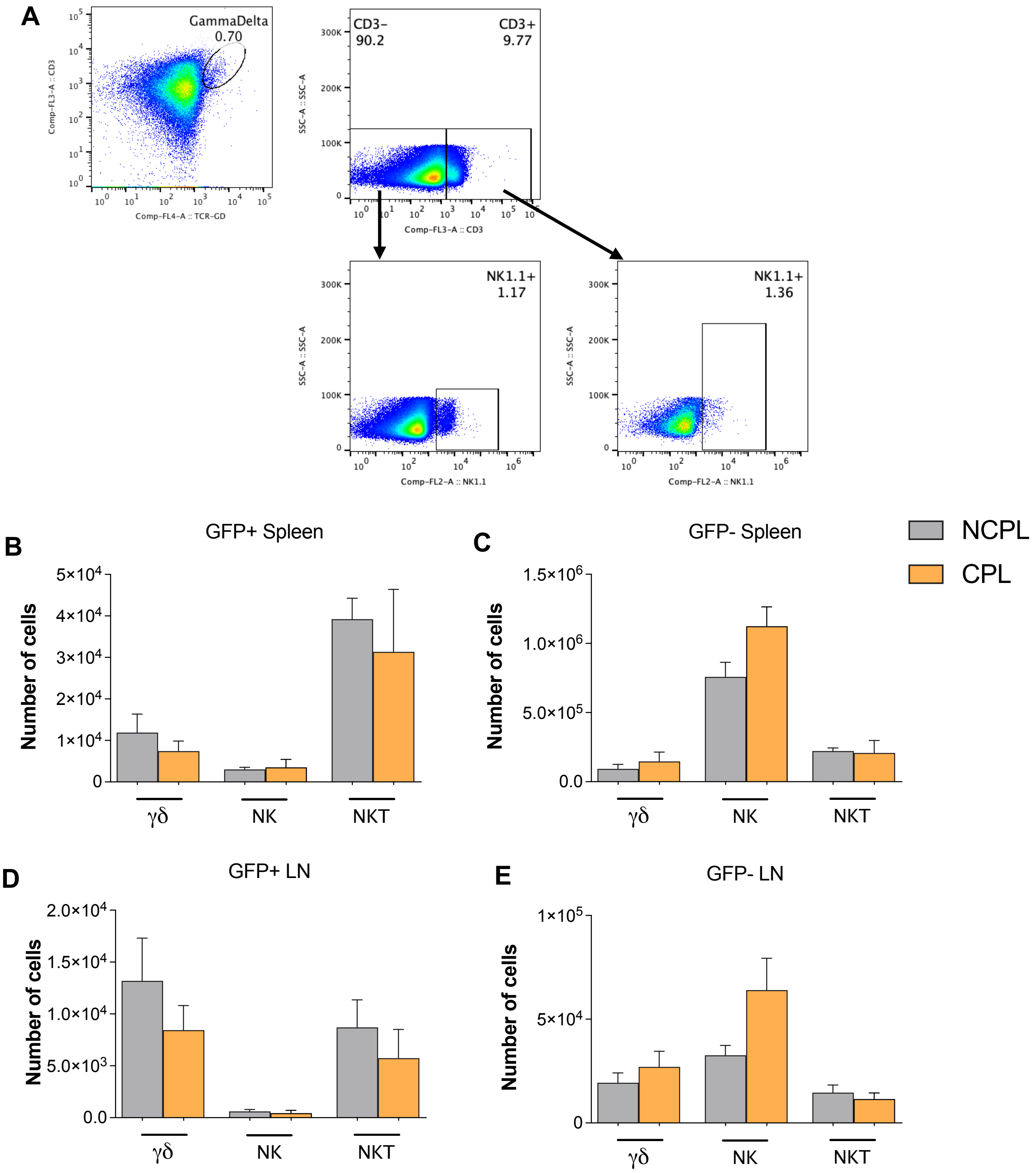

Supplementary Figure 3 Legend

(A) Flow cytometry plots show the gating strategy to identify $\gamma\delta$T-cells (CD3+$\gamma\delta$+), NK (CD3-NK+) and NKT-cells (CD3+NK+) after gating on live cells.

(B-E) Bar charts show number of $\gamma\delta$T-cells, NK and NKT-cells in spleen and LN gated on either GFP+ or GFP- cells.
